# SERS-Tags: Selective Immobilization and Detection of Bacteria by Strain-Specific Antibodies and Surface-Enhanced Raman Scattering

**DOI:** 10.3390/bios13020182

**Published:** 2023-01-24

**Authors:** Markéta Benešová, Silvie Bernatová, Filip Mika, Zuzana Pokorná, Jan Ježek, Martin Šiler, Ota Samek, Filip Růžička, Katarina Rebrošová, Pavel Zemánek, Zdeněk Pilát

**Affiliations:** 1 Institute of Scientific Instruments of the Czech Academy of Sciences, v.v.i., Královopolská 147, 612 64 Brno, Czech Republic; 2 Department of Microbiology, Faculty of Medicine of Masaryk University and St. Anne’s University Hospital, Pekařská 53, 656 91 Brno, Czech Republic

**Keywords:** SERS-tag, *Escherichia coli*, sandwich immunoassay, single-cell detection

## Abstract

Efficient separation and sensitive identification of pathogenic bacterial strains is essential for a prosperous modern society, with direct applications in medical diagnostics, drug discovery, biodefense, and food safety. We developed a fast and reliable method for antibody-based selective immobilization of bacteria from suspension onto a gold-plated glass surface, followed by detection using strain-specific antibodies linked to gold nanoparticles decorated with a reporter molecule. The reporter molecules are subsequently detected by surface-enhanced Raman spectroscopy (SERS). Such a multi-functionalized nanoparticle is called a SERS-tag. The presented procedure uses widely accessible and cheap materials for manufacturing and functionalization of the nanoparticles and the immobilization surfaces. Here, we exemplify the use of the produced SERS-tags for sensitive single-cell detection of opportunistic pathogen *Escherichia coli,* and we demonstrate the selectivity of our method using two other bacterial strains, *Staphylococcus aureus* and *Serratia marcescens*, as negative controls. We believe that the described approach has a potential to inspire the development of novel medical diagnostic tools for rapid identification of bacterial pathogens.

## 1. Introduction

One of the crucial tasks in clinical practice is to fight against pathogenic microorganisms. Treatment of infections is frequently complicated by the resistance of microorganisms to antibiotics, and the supply of new antibiotics is limited. The effective lifetime of an antibiotic can be extended by avoiding overuse of broad-spectrum antibiotics with improved diagnostics of clinically relevant pathogens [1]. For example, the frequent and serious infections caused by the widely distributed opportunistic pathogen *Escherichia coli* (*E. coli*), an inhabitant of the human and animal digestive tract, are usually detected by cultivation methods, biochemical identification, matrix-assisted laser desorption/ionization-time of flight (MALDI-TOF) mass spectroscopy [2,3], polymerase chain reaction (PCR), or with antibodies [4]. However, MALDI-TOF, cultivation and colony-counting methods are inconvenient, given that fast pathogen detection is particularly important to allow timely intervention [5,6]. While PCR and antibody-based methods do not strictly require overnight cultivation, they still have sensitivity limitations and take several hours, while the patient may be in danger of fatal complications such as sepsis and anaphylactic shock.

Rapid detection, sensitive discrimination, and efficient elimination of pathogens represent important research goals that require an effective interdisciplinary cooperation between microbiology and pharmacology, as well as applied physics and nanotechnology. The applications of new pathogen detection techniques are not limited to medical diagnostics, but include also food safety, biodefense, and drug discovery [1,5,7].

Raman spectroscopy (RS) is considered an effective alternative method for the identification of pathogenic bacteria, which has been repeatedly demonstrated [8,9,10,11,12]. The main drawback of RS is its low sensitivity, causing prolonged measurement time or even inability to effectively detect the pathogen. Improvement of the signal intensity can be achieved by advanced RS modifications such as surface enhanced Raman spectroscopy (SERS) that allows Raman signal amplification by several orders of magnitude due to plasmonic features of metal nanoparticles or nanostructured surfaces. The plasmonic properties of the nanostructure must be tuned for a particular laser wavelength to effectively excite a localized surface plasmon polariton (LSPR). The enhancement is provided by the interaction of the localized plasmon oscillation with the valence electrons of the studied molecule and can reach 10^3^–10^14^ [13,14,15], which is sufficient to detect even femtomolar amounts of biomolecules [14]. The short radiation range of hot-spots (below 5 nm) presents a significant disadvantage in the direct use of SERS for identification of bacteria, since the majority of SERS signals from the bacteria attached to the SERS substrate originate only from the cell wall. Inter-species differences in the cell wall composition are usually insufficient for reliable identification of the bacterial pathogen. An efficient solution of this obstacle is to use specific Raman-active labels that are selectively attached to the bacterial cells [7,16].

Optical labels are essential for probing specific target molecules in complex biological systems. For example, quantum dots are widely used as fluorescent bio-imaging labels [6,17], but their cytotoxicity may present an obstacle for many biomedical applications. In cases where fluorescence-based labels are problematic, modified gold (Au) nanoparticles may be used as an alternative due to their biocompatibility, high electron density, water solubility, and stability. SERS-labels represent an ideal method for identification of bacteria, allowing rapid, noncontact detection of individual bacterial cells in a small sample volume. SERS-tags are nanoprobes based on noble metal nanoparticles, decorated with reporter molecules and antibodies, which serve as labels for detection of specific antigens in complex biological systems [18,19,20,21,22]. Very often the probes are combined with nanoparticle-based magnetic separation for tandem immobilization and detection of pathogens [18,19,20,23]. The possibility to employ multiple different Raman reporters on SERS-tags with different antibodies allows simultaneous detection of multiple bacterial species [7,24].

Au nanorods (AuNRs) are ideal candidates for production of SERS-tags, because of the two absorption bands exhibited by the surface plasmons resonant along the two main axes of the AuNR: the longitudinal axis and the transversal axis [20,25]. The dimensions of the AuNRs along their axes are related to the excitation of surface plasmons at two different wavelengths: longitudinal surface plasmon wavelength (LSPW) and transversal surface plasmon wavelength (TSPW), which can be tuned by the synthetic process [26,27,28,29]. By changing the longitudinal dimension of the AuNR, the LSPW band can be tuned from the visible to near infra-red region [30,31].

**Figure 1:**
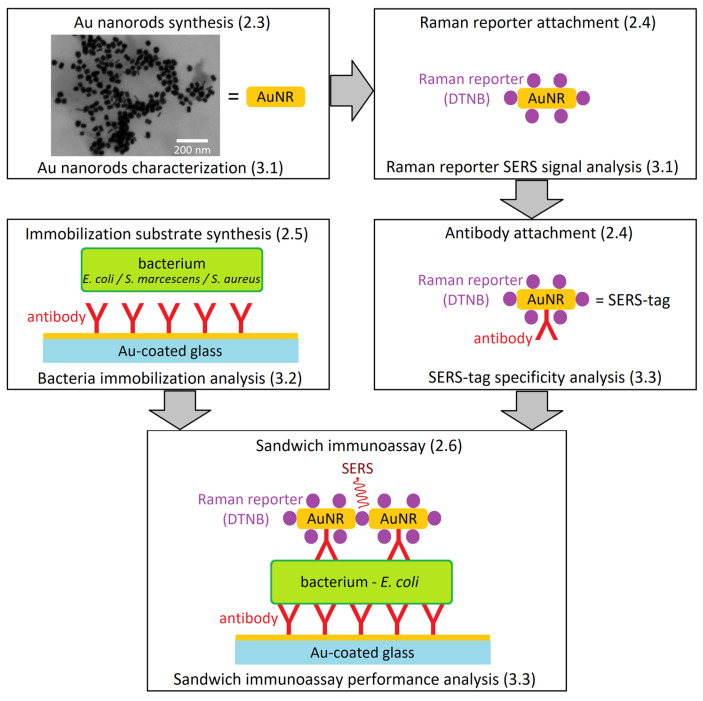
Work-flow diagram leading to the sandwich immunoassay for *E. coli* detection with SERS-tags. (Schematic depiction—parts not to scale.) The numbers in parentheses refer to the article sections dealing with methodology (2.x) and results (3.x) of the individual steps. The synthesis of AuNRs is followed by their characterization with electron microscopy. Reporter molecules are then attached to the AuNRs and their SERS signal is analyzed. Subsequent antibody attachment provides a complete SERS-tag. Au-coated glass is derivatized with antibodies and the immobilization of bacteria is tested. In the final step, the branches of the diagram unite to afford the sandwich immunoassay, and its analytical performance is tested with multiple bacterial species.

Here, we present so-called sandwich immunoassay for *E. coli* detection by a combination of initial anti-*E. coli*-antibody-based immobilization of *E. coli* cells on a Au-covered glass chip, followed by a selective tagging of the immobilized *E. coli* with the SERS-tag prepared from AuNRs surface-modified by an anti-*E. coli* antibody and a Raman reporter. Molecule-specific Raman spectra of the Raman reporter then allow specific detection of the presence of *E. coli*, the target bacterium, on a single cell level. To demonstrate the specificity of our approach, *Staphylococcus aureus* and *Serratia marcescens* were used as negative controls. The synthesized AuNRs were observed by electron microscopy, their dimensions were characterized, and their amplification of the Raman reporter signal was quantified using enhancement factors. The selectivity of the immobilization procedure and SERS-tag binding was calculated, and the capacity of the sandwich immunoassay to reliably detect *E. coli* was discussed. The complete work-flow is schematized on Figure 1.

The biological application of SERS using functionalized nanoparticles was reported previously [22], and the sandwich strategy has also been already reported [32]. However, our sandwich strategy is not employed to enhance the SERS signal intensity as such. Instead, it enables antibody-based immobilization of the selected bacteria (*E. coli*) even from a diluted sample and serves as an effective pre-selection for the subsequent step, since the SERS-tags carry the identical antibody. Therefore, even if the immobilization yields some proportion of unwanted cells (by non-specific interactions), these will not interact with enough SERS-tags to produce a SERS signal comparable to *E. coli*. Moreover, while the detection of *E. coli* by SERS was already reported [21,23], our SERS-tag-based approach does not rely on the SERS spectrum from the bacterium itself, but from a Raman reporter molecule, which is a part of the SERS-tag and its signal is exceptionally strong, allowing reliable and fast detection on a single cell level. Moreover, our approach can be modified to detect virtually any microorganism, or virus, without prior knowledge of its intrinsic SERS spectrum, simply by choosing the appropriate antibody for the SERS-tag and the immobilization surface syntheses.

## 2. Experimental Section

### 2.1. Materials and Reagents

Biotin-conjugated anti-*E. coli* rabbit polyclonal (IgG) antibody was obtained from Abcam plc. (Cambridge, UK). This antibody is specific for many “O” and “K” antigenic serotypes of *E. coli*. Antiserum is not absorbed and does cross-react with bacteria from related family *Enterobacteriaceae*. Avidin (immunopure), sodium borohydride (NaBH_4_), bovine serum albumin (BSA), 5,5′-dithiobis-2-nitrobenzoic acid (DTNB), hexadecyltrimethylammonium bromide (CTAB), hydrogen tetrachloroaurate (HAuCl_4_), 11-mercaptoundecanoic acid (11-MUA), 3-mercaptopropionic acid (3-MPA), *N*-(3-dimethylaminopropyl)-*N*′-ethylcarbodiimide hydrochloride (EDC), and phosphate-buffered saline (PBS) were obtained from Sigma-Aldrich (Munich, Germany). Tween 20 and 2-(*N*-Morpholino)-ethane sulphonic acid (MES buffer) were obtained from Carl Roth (Karlsruhe, Germany). *N*-hydroxysuccinimide sodium salt (NHS) was obtained from Fluka Analytical (Munich, Germany). Silver nitrate (AgNO_3_), sodium hydroxide (NaOH), L-ascorbic acid (AA), ethanolamine and absolute ethanol (abs. EtOH) were obtained from PENTA (Chrudim, Czech Republic). All the chemicals were analytically pure and were used as received. Deionized H_2_O (dH_2_O) used for preparing all solutions was prepared in a standard commercial purification system with resistivity about 5 MΩ/cm^2^.

### 2.2. Microorganisms

*Escherichia coli* K-12 was obtained from Prof. Ute Neugebauer, University clinic in Jena, Germany. *Staphylococcus aureus* CCM 4890 was obtained from Prof. Filip Růžička, St. Anne’s University Hospital Brno, Czech Republic. *Serratia marcescens* CCM 8587 was obtained from Czech Collection of Microorganisms, Masaryk University, in Brno, Czech Republic. The bacteria were cultivated on Mueller–Hinton agar obtained from Sigma-Aldrich (St. Louis, MO, USA) at 37 °C for 18 h. Samples were prepared by suspending a single colony of the bacteria in 1 mL of 0.1 M PBS at pH 7.4.

### 2.3. Fabrication of Gold Nanorods

Gold nanorods (AuNRs) were prepared by a modified seed-mediated growth technique inspired by Tamer et al. [7]. The synthesis was optimized to yield AuNRs with LSPW tuned to 785 nm excitation wavelength. First, 7.5 mL of 0.1 M CTAB and 250 µL of 0.01 M HAuCl_4_ in dH_2_O were mixed. Then, 600 µL of 0.01 M ice-cold NaBH_4_ in dH_2_O with 100 µL of 0.1 M NaOH was added rapidly and the seed solution was left for 5 min at room temperature (RT) to turn dark brown. To prepare AuNRs, the solutions of reagents in dH_2_O were mixed in the given order: 1 mL of 0.01 M HAuCl_4_ was added to 4.75 mL of 0.1 M CTAB, followed by 60 µL of 4 × 10^−3^ M AgNO**_3_**. The solution turned brownish-yellow. To this mixture, 250 µL of 0.1 M AA in dH_2_O was added dropwise. The resulting stock solution turned colorless. Subsequently, the stock solution was mixed with 5 µL seed solution, followed by a few seconds of stirring and incubation for 3 h at RT to yield the AuNRs, turning the solution dark purple. The AuNRs were collected by centrifugation at 6700× *g* for 10 min, the supernatant was removed and the AuNRs resuspended in dH_2_O. The washing was repeated 2 times.

### 2.4. Fabrication of SERS-Tags

The obtained AuNRs were immersed in 1 mL of 0.1 mM DTNB [33] in abs. EtOH overnight to form a self-assembled monolayer (SAM). Subsequently, the AuNRs were modified with a mixture of 100 mM 3-MPA and 150 mM 11-MUA in 1 mL of abs. EtOH for 18 h at RT. AuNRs were collected by centrifugation at 6700× *g* for 10 min and resuspended in 70% EtOH in an ultrasonic bath for 30 s; washing was repeated 3 times. Then, AuNRs were immersed for 35 min in 0.05 M NHS and 0.2 M EDC in 0.1 M MES buffer at pH 6.5, centrifuged at 6700× *g* for 10 min and the supernatant was removed. AuNRs were resuspended in 0.5 mg/mL avidin in MES buffer and incubated for 30 min at RT, collected by centrifugation at 6700× *g* for 10 min, and resuspended in MES buffer in an ultrasonic bath for 30 s. Washing was repeated 3 times to remove unreacted avidin. To block nonspecific interactions, 1 mL of 10% (*v*/*v*) ethanolamine in MES buffer was added and the mixture incubated for 1 h at RT. 90 µL of 0.1 mg/mL biotinylated antibody was added and the mixture was left for >2 h at RT [24]. AuNRs were centrifuged at 6700× *g* for 10 min, washed and resuspended in PBST buffer (0.1 M PBS at pH 7.4 with 0.5% Tween) in ultrasonic bath for 30 s; washing was repeated for 3 times. 1 mg/mL of BSA in 0.1 M PBS buffer at pH 7.4 was added and the mixture was incubated for 4 h at RT [34]. AuNRs were collected by centrifugation at 6700× *g* for 10 min, washed and resuspended in PBST buffer in an ultrasonic bath for 30 s; the process was repeated 3 times. After the final wash, AuNRs were resuspended in 0.1 M PBS buffer and stored at 4 °C [35].

### 2.5. Fabrication of Gold-Coated Slides

Standard 76 mm × 26 mm glass microscope slides were cut to 1 cm wide segments and coated in a sputter coater Q150T ES (Quorum Technologies, Lewes, UK) with 100 nm layer of chromium to improve adhesion and subsequently with 100 nm layer of gold. Coatings of substantially different thickness were found to be unstable, prone to detachment. Au-coated slides were modified with 100 mM 3-MPA and 150 mM 11-MUA in abs. EtOH for 18 h at RT, washed three times with 2 mL of 70% EtOH, and immersed for 35 min in 0.05 M NHS and 0.2 M EDC solution prepared in 0.1 M MES buffer, followed by washing 3 times with 2 mL of MES buffer. 0.5 mg/mL avidin in MES buffer was added for 30 min, followed by washing 3 times with 2 mL of MES buffer. 1% (*v*/*v*) ethanolamine in MES buffer was applied for 1 h at RT. 0.1 mg/mL biotinylated antibody was added and the slides were left for 2 h at RT, followed by washing 3 times with 2 mL of PBST buffer and treatment with 2 mg/mL of BSA in PBS buffer for 4 h at RT, followed by washing 3 times with 2 mL of PBST buffer. After the final wash, the slides were immersed in PBS and stored in the refrigerator for further use.

### 2.6. Sandwich Immunoassay

The sandwich immunoassay is schematically described in Figure 1. A suspension of *E. coli* was prepared by suspending one bacterial colony in 1 mL PBS buffer (about 1 × 10^5^ CFU/mL), followed by centrifugation 6700× *g* for 2 min, supernatant removal and resuspension in 1 mL of 0.1 M PBS buffer. Au-coated slides derivatized with the antibody were then immersed in the bacterial suspension for 30 min, followed by washing with 2 mL PBS buffer for 3 times, 2 mL PBST solution (mixture of 0.1 M PBS buffer at pH 7.4 and 0.5% Tween) for 3 times and 2 mL PBS for 3 times. Then, AuNRs were applied by immersing the Au-coated slides into the AuNR suspension for 30 min. After incubation, the washing process was repeated and followed by SERS measurement. The cross-specificity of the immunoassay was tested using the identical protocol with *S. marcescens* CCM 8587 and *S. aureus* CCM 4890 strains. The resulting SERS signal intensities were compared to the data obtained for *E. coli.* SERS measurement parameters (cf. Section 2.8).

### 2.7. Electron Microscopy

Scanning transmission electron microscopy (STEM) of AuNRs was performed on a scanning electron microscope Helios 5 Hydra UX DualBeam (Thermo Fisher Scientific, Brno, Czech Republic) in Bright Field mode and High Angle Annular Dark Field mode, with 30 kV acceleration voltage, 50 pA current, 4.7 mm working distance and 200,000× magnification. Samples for STEM were prepared by pipetting 3 µL of nanoparticle solution onto STEM copper grids and allowing them to dry for 10 min.

### 2.8. Raman Spectroscopy

Raman spectra were acquired with commercial instrument InVia (Renishaw, Gloucester, UK), with laser wavelength 785 nm and laser power 14.5 mW (before the objective lens). Two microscope objectives, 50×, NA = 0.75, and 20×, NA = 0.4 (N PLAN EPI, Leica, Wetzlar, Germany) were used as specified in Section 3 for different results. The focal spot of the objective with 50× magnification was 2 μm × 5 μm. Raman spectra in the range 614–1724 cm^−1^ were collected with integration time 10 s. SERS and RS were always measured with identical microscope objective and acquisition parameters. Before each acquisition, the excitation laser was refocused on the sample surface. We performed 6–15 measurements at different locations on the sample surface. The spectral data were processed and analyzed in homemade software written in Matlab (MathWorks, Natick, MA, USA) along with MS Excel (Microsoft, Washington, DC, USA). To remove fluorescence from the Raman spectra, we used rolling-circle filter [36] with circle diameter 1000 pixels, and 300 pixels, 10 passes each (1 pixel corresponding to approx. 1 cm^−1^). To remove high frequency noise, we used a Savitzky–Golay filter with 5 passes, order 2, and frame length 7 pixels. Peak positions were determined with precision ± 1 cm^−1^. The spectroscopic data are available in the RamanBase repository at https://ramanbase.org/doi/ (accessed on 19 January 2023) with the DOI of this article added after the last slash.

## 3. Results and Discussion

### 3.1. AuNRs—SERS Substrate Characterization

**Figure 2:**
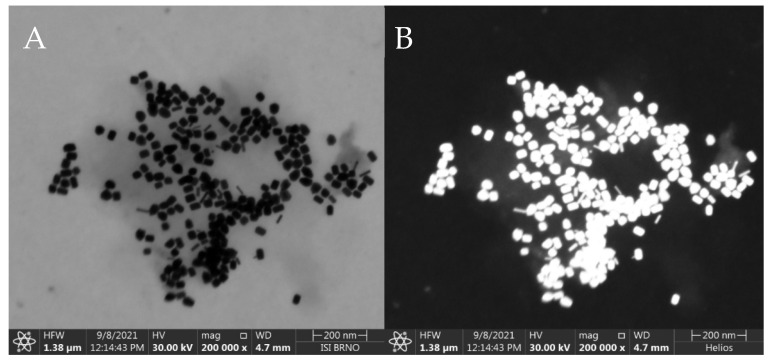
SEM image of the synthesized AuNRs placed on a thin carbon film. Taken at 30 kV, 50 pA with transmission detector in (**A**): Bright Field, (**B**): High Angle Annular Dark Field mode. Instrument: Helios G4 (Courtesy of Thermo Fisher Scientific). Average dimensions: (≈ 90%): 40 ± 4 nm × 24 ± 2 nm, (≈ 10%): 42 ± 4 nm × 8 ± 2 nm. Measurement parameters cf. 2.7.

**Figure 3:**
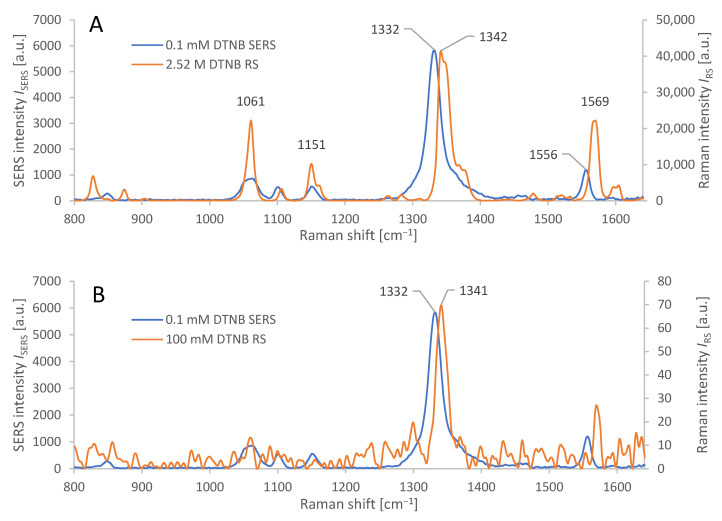
(**A**): Characteristic spectral response from AuNR-based SERS of adsorbed DTNB, applied in concentration 0.1 mM, compared to Raman spectroscopy of pure solid DTNB (2.52 M). Average spectra from 6 measurements each for SERS and RS. Absolute values of the spectral response were measured at identical laser power and integration time. (**B**)**:** Characteristic spectral response from AuNR based SERS of adsorbed DTNB, applied in concentration 0.1 mM, compared to Raman spectroscopy of a thin layer of DTNB on CaF_2_, from 100 mM solution, cf. Section 3.1. Average spectra from 6 measurements (SERS) and 15 measurements (RS). Microscope objective: 20×; for measurement parameters, see Section 2.8.

The synthesized AuNRs were observed by STEM and the resulting images were used for manual calculation of their average dimensions. The AuNRs were found to be a mixture consisting of a dominant fraction—short and thick AuNRs (average dimensions 40 ± 4 nm by 24 ± 2 nm) and a smaller fraction (≈ 10%) of thinner, longer AuNRs (average dimensions 42 ± 4 nm by 8 ± 2 nm), see Figure 2. The synthetic protocol repeatedly afforded AuNRs with such size distribution, which were used for the following steps as such, to serve as the SERS substrate for the SERS-tag. The reporter molecule was introduced to the SERS substrate by incubation of the AuNRs with DTNB solution, which afforded a self-assembled monolayer (SAM) consisting of 2-nitro-5-mercaptobenzoic acids (TNB) molecules resulting from homolytic cleavage of the S-S bond of DTNB [37]. Both Raman and SERS spectra are presented in Figure 3A. The 10 cm^−1^ difference between the maxima of the dominant Raman signal of pure DTNB (1342 cm^−1^) and the dominant signal from the SERS-tag (1332 cm^−1^) may be attributed to the cleavage of this S-S bond and creation of a Au-S bond. Another factor may be the changes on the carboxyl group due to the derivatization process for the attachment of antibodies. When adsorbed on AuNRs, DTNB is used to refer to 2 molar equivalents of TNB.

We calculated the analytical enhancement factor (AEF) of the AuNR-based SERS substrate (with DTNB as the analyte) from relation (1) [38]:

(1) $$\mathrm{AEF}=\frac{I_{\mathrm{SERS}}/c_{\mathrm{SERS}}}{I_{\mathrm{RS}}/c_{\mathrm{RS}}}$$

where *I*_RS_ represents Raman signal intensity and *I*_SERS_ represents SERS signal intensity from samples containing DTNB in molar concentrations *c*_RS_ and *c*_SERS_ in the respective samples [38].

The input variables to the equation (1) are the following: the average intensity (*I*_SERS_) of the dominant peak (1332 cm^−1^) in the SERS spectrum of DTNB adsorbed on the AuNRs (from a solution with molar concentration *c*_SERS_ = 0.1 mmol/kg), and the average intensity (*I*_RS_) of the dominant peak (1342 cm^−1^) in the conventional Raman spectrum of pure DTNB (with molar concentration *c*_RS_ = 2.52 mol/kg), measured under identical conditions, (cf. Section 2.8, 20× objective). The ratios *I*_RS_/*c*_RS_ (and *I*_SERS_/*c*_SERS_) represent the Raman (and SERS) signal intensities per mole of DTNB in the sample. The ratio of these values represents the analytical enhancement factor of our SERS structures: AEF ≈ 3500 ± 500.

To further characterize the enhancement of AuNR-based SERS substrate with regards to the amount of molecules in the scattering volume, we calculated the SERS enhancement factor (SERS EF), by relation (2) [38]:

(2) $$\mathrm{SERS}\;\mathrm{EF}=\frac{I_{\mathrm{SERS}}/N_{\mathrm{Surf}}}{I_{\mathrm{RS}}/N_{\mathrm{Vol}}}$$

where *N*_Vol_ *= c*_RS_*V* is the average molar amount of DTNB molecules in the scattering volume *V* for the conventional Raman measurement. For the measurement of *I***_RS_**, we used a thin film of DTNB from made by evaporating 1 µL of *c*_RS_ = 100 mM DTNB solution in abs. EtOH on a polished CaF_2_ slab, where it spread to a circular spot, 6 mm in diameter. Scattering volume *V* is defined by the focal spot area and DTNB film thickness, which is impractical to measure, so *N*_Vol_ was instead, determined by relation *N*_Vol_ *= a*_F_ (*c*_RS_*V*_Sol_/*a*_S_), where *c*_RS_ is the molar concentration of DTNB solution, *V*_Sol_ is its volume, *a*_S_ is the total area of the spot, and *a*_F_ is the focal spot area (10 µm^2^). *N*_Surf_ denotes the average number of adsorbed molecules in the scattering volume for the SERS measurement [38]. The calculation of *N*_Surf_ for the SERS experiment was based on the number of moles of DTNB, spread over the total surface area (*a*_Tot_) of the AuNRs. This was calculated from the total weight of gold used in AuNR synthesis (*m*_Tot_) (cf. Section 2.3) and the calculated average weight (*m*_NR_) and area (*a*_NR_) of each AuNR, calculated manually from the STEM images and density of Au, by the following relation: *a*_Tot_ = *a*_NR_ (*m*_Tot/_*m*_NR_). The molar amount of DTNB (*N*_Surf_) adsorbed on the AuNRs was calculated from the volume *V*_Sol_ (1 mL) and concentration *c*_SERS_ (0.1 mM) used in the synthesis of Raman tags (cf. Section 2.4), by a relation: *N*_Surf_ = *a*_F_ (*c*_SERS_*V*_Sol_/*a*_Tot_). The incomplete adsorption of DTNB on AuNRs, as well as the losses of Au during AuNR synthesis were neglected. In principle, these errors partially compensate each other. SERS was measured on nanoparticles that were allowed to settle into a monolayer. The average Raman and SERS spectra are depicted in Figure 3B; acquisition parameters were identical (cf. Section 2.8, 20× objective). The ratios *I*_RS_/*N*_Vol_ (and *I*_SERS_/*N*_Surf_) represent the Raman (and SERS) signal intensities normalized to the molar amount of DNTB in the scattering volume. Their ratio (relation 2) represents the SERS enhancement factor, reaching the following value: SERS EF ≈ 100,000 ± 10,000.

### 3.2. Selective Immunochemical Immobilization of Bacteria on Au-Coated Slide

**Figure 4:**
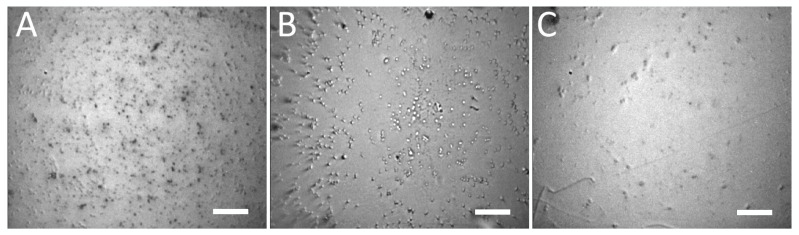
Bright-field microscope images of Au-coated glass derivatized with *E. coli*-specific antibody and treated with suspensions containing *E. coli*; >1700 cells/FOV (**A**), *S. aureus*; ≈ 400 cells/FOV (**B**) and *S. marcescens*; ≈ 100 cells/FOV (**C**). The applied suspensions had equivalent bacterial concentrations in the order 1 × 10^5^ CFU/mL. The Au-coated glass was washed several times with PBS before observation, cf. Section 2.6. Microscope objective: 20×; scale bars: 20 µm.

Prior to the actual detection, we selectively immobilized the bacteria on Au-coated glass slide with *E. coli*-specific antibody. The surface was treated with suspensions containing *E. coli*, *S. aureus*, and *S. marcescens*. The typical result of the immobilization can be seen on Figure 4. The obtained selectivity was such that the concentration of *E. coli* (specific interaction) was ≈ 1700 ± 200 cells per field of view (FOV), while the concentrations of *S. aureus* and *S. marcescens* (non-specific interaction) were ≈ 400 ± 50 cells per FOV and ≈ 100 ± 10 cells per FOV, respectively. This makes the specific interaction at least 4×, but up to 14× more effective for the immobilization, than the non-specific interactions with bacteria other than *E. coli*. We attribute the high number of non-specific interactions with *S. aureus* to the fact that it may produce copious extracellular matter, which might improve its adhesion to various surfaces. Despite this tendency to attach to surfaces by non-specific mechanisms, this was not observed in all the experimental runs; in some instances, the non-specific binding was nearly absent, leaving less than 10 cells per FOV. Moreover, with regards to the SERS-tags, the non-specific binding of *S. aureus* was minimal, with more non-specific binding to SERS-tags being observed with *S. marcescens* (see the following section).

### 3.3. Detection of E. coli Using SERS-Tags in the Sandwich Immunoassay

**Figure 5:**
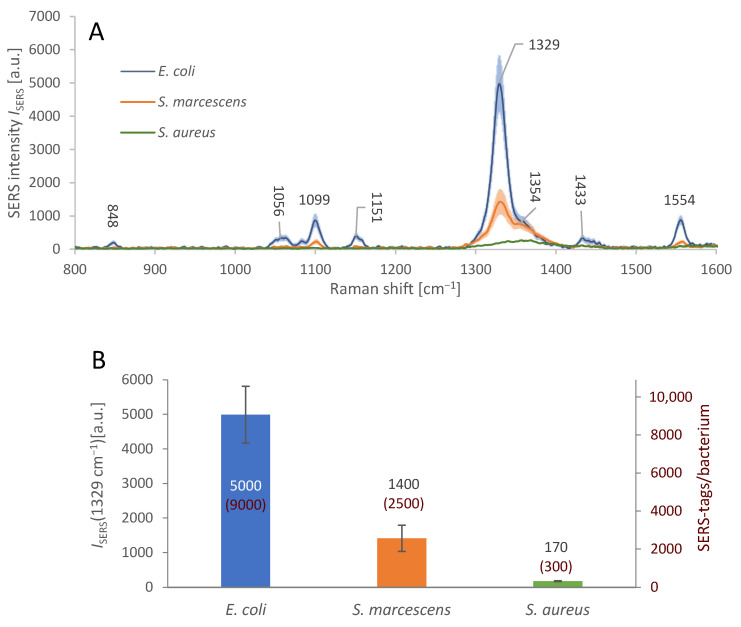
Sandwich immunoassay SERS signal intensity and specificity. (**A**): Average SERS spectra from individual cells of different bacterial species (see legend), in the *E. coli*-specific sandwich complex. *E. coli* cells were covered with the SERS-tags and detected by the strong SERS signal of DTNB. In case of *S. marcescens* and *S. aureus*, the signal is weaker, indicating relatively low level of undesirable non-specific interactions between these bacteria and the SERS-tags. The nominal SERS signal intensities are presented in the bar graph below. The shaded areas represent the 95% confidence interval. Microscope objective: 50×. (**B**): Average SERS signal intensity maxima at 1329 cm^−1^, from the bacteria subjected to sandwich immunoassay, i.e., immobilization on the gold-plated glass covered in antibodies and to the detection process involving SERS-tags. The positive, specific signal of *E. coli* is more than 3.5× stronger than the non-specific signal of the *S. marcescens* and over 28× stronger than that of *S. aureus*. The numbers in red show the calculated amounts of the individual SERS-tags per bacterium. The error bars represent the 95% confidence interval of *I*_SERS_.

The testing of AuNR-based SERS substrate parameters and the immobilization process was followed by merging both these elements together to realize the sandwich immunoassay, cf. Section 2.6. Figure 5A shows the average spectral response of the immobilized bacteria with attached SERS-tags. The density of the SERS-tags attached on the bacterial surface was different, judging from the differences in the average SERS signal intensity at 1329 cm^−1^; the maximum of the peak is shifted by 3 cm^−1^ in comparison with measurements on SERS-tags pre-application. The positive, specific signal of *E. coli* is more than 3.5× stronger than the non-specific signal of *S. marcescens* and over 28× stronger than that of *S. aureus*.

This level of specific response to *E. coli*, relative to the non-specific interactions, is clearly distinguishable and sufficient for detection of the target bacterium in an isolated bacterial culture containing a single species. However, the spurious signal from *S. marcescens* may be misrepresented as a positive response in a mixed culture, especially with multiple bacteria in similar amounts. This drawback can be overcome by calculating the average number of SERS-tags per bacterium, since only in positive detections we found the bacterial surface about 90% covered with SERS-tags, while in nonspecific reactions, SERS-tags were always occupying less than 25% of the bacterial surface. We calculated the average number of SERS-tags attached on the surface of each bacterium for the three bacterial species, based on the observed average Raman intensity *I*_SERS_ at 1329 cm^−1^. We used the observed average dimensions of each AuNR to calculate the maximal amount *N*_max_ of the AuNRs to fit into the focal area of the used 50× objective (*N*_max_ ≈ 1000 ± 100 AuNRs/µm^2^). We used *I*_SERS_*/N*_max_ ratio as the typical signal intensity of a single SERS-tag, which served to derive the approximate number of SERS-tags per cell from average I_SERS_ obtained for *E. coli*, *S. marcescens*, and *S. aureus* (see Figure 5B). We also calculated the surface area of *E. coli* cell by approximation to a cylinder 1 µm wide and 2.5 µm long [39], amounting to ≈ 9.4 µm^2^, with maximal amount of the adsorbed SERS-tags per single bacterium equal to ≈ 9000 ± 1000. This calculation is based on the assumption that the SERS-tags attach to the bacterium with the largest surface area possible, since the antibodies make them “stick” to the antigens on the bacterial surface, with random movements forming more attachment points with high probability. The surface area of *S. marcescens* is roughly 6 µm^2^, (0.8 µm in diameter, 2 µm length) [40], and *S. aureus* surface area is about 7 µm^2^, (1.5 µm diameter) [41]. The relative amount of SERS-tag coverage of each bacterium was calculated: *E. coli* 90% (± 5%), *S. marcescens* 25% (± 5%), and *S. aureus* 3% (± 1%).

## 4. Conclusions

We describe a comprehensive universal protocol for preparation of AuNRs, SERS-tags, and selective immobilization substrates from easily accessible materials, collectively forming a sandwich immunoassay for detection of selected bacterial species. The realized sandwich immunoassay allowed us to selectively immobilize *E. coli* cells based on antigen-antibody affinity and to detect *E. coli* on the level of individual cells by a selective attachment of SERS-tags. Our pilot study suggests that this approach is useful for detection of a selected bacterial species from a pure culture and probably even from mixtures, given the possibility to focus on individual cells. The average relative occupancy of the surface area of each *E. coli* cell by the SERS-tags was calculated to be over 90% (± 5%). This information can be useful for assessment of the specificity of the detection, since the non-specific binding reached an occupancy of only 25% (± 5%).

The unique arrangement of our SERS-tag nanoprobes, schematized in Figure 1, allows sensitive, selective and rapid identification of bacteria. The described sandwich immunoassay can be easily implemented on a microfluidic platform, allowing automation of the analytical procedure. Moreover, using several SERS-tags with different antibodies and reporter molecules allows simultaneous identification of multiple bacterial species in the studied sample.

## Data Availability

All the data presented in this study are available on request from the corresponding author. The raw spectroscopic data are available at https://ramanbase.org/doi/ (accessed on 19 January 2023) with the DOI of this article added after the last slash.

## Abbreviations

The following abbreviations are used in this manuscript:

3-MPA	3-mercaptopropionic acid
11-MUA	11-mercaptoundecanoic acid
AA	L-ascorbic acid
abs. EtOH	Absolute ethanol
AEF	Analytical enhancement factor
*a*_F_	Focal spot area
Ag	Silver
AgNO_3_	Silver nitrate
*a*_NR_	Area of each AuNR
*a*_S_	Total area of the spot
*a*_Tot_	Total surface area
Au	Gold
AuNRs	Gold nanorods
BSA	Bovine serum albumin
dH_2_O	Deionized H_2_O
*c*_SERS_	Molar concentrations—SERS
*c*_RS_	Molar concentrations—Raman spectroscopy
CFU	Colony-forming unit
DTNB	5,5′-dithiobis-2-nitrobenzoic acid
EDC	*N*-(3-dimethylaminopropyl)-*N*′-ethylcarbodiimide hydrochloride
*E. coli*	*Escherichia coli* K-12
FOV	Field of view
HAuCl_4_	Hydrogen tetrachloroaurate
*I*_RS_	Raman signal intensity
*I*_SERS_	SERS signal intensity
LSPR	Localized surface plasmon resonance
LSPW	Longitudinal surface plasmon wavelength
MALDI-TOF	Matrix-assisted laser desorption/ionization-time of flight
MES buffer	2-(*N*-Morpholino)-ethane sulphonic acid
*m*_NR_	Calculated average weight
MS	Mass spectrometry
*m*_Tot_	Total weight of gold used in AuNR synthesis
NA	Numerical aperture
NHS	*N*-hydroxysuccinimide sodium salt
NIR	Near infrared
*N*_Surf_	Average number of adsorbed molecules for the SERS measurement
*N*_Vol_	Average number of molecules
PBS	Phosphate-buffered saline
PCR	Polymerase chain reaction
RT	Room temperature
RS	Raman spectroscopy
*S. aureus*	*Staphylococcus aureus*
*S. marcescens*	*Serratia marcescens* CCM 8587
SAM	Self-assembled monolayer
SERS	Surface-enhanced Raman spectroscopy
SERS EF	SERS enhancement factor
STEM	Scanning transmission electron microscopy
TNB	2-nitro-5-mercaptbenzoic acid
TSPW	Transversal surface plasmon wavelength
